# Stepwise shortening of agalsidase beta infusion duration in Fabry disease: Clinical experience with infusion rate escalation protocol

**DOI:** 10.1002/mgg3.1659

**Published:** 2021-03-23

**Authors:** Eleonora Riccio, Mario Zanfardino, Monica Franzese, Ivana Capuano, Pasquale Buonanno, Lucia Ferreri, Maria Amicone, Antonio Pisani

**Affiliations:** ^1^ Institute for Biomedical Research and Innovation National Research Council of Italy Palermo Italy; ^2^ IRCCS SDN Naples Italy; ^3^ Department of Public Health, Chair of Nephrology University Federico II of Naples Naples Italy; ^4^ Department of Neurosciences, Reproductive and Odontostomatological Sciences University of Naples Federico II Naples Italy

**Keywords:** agalsidase beta, enzyme replacement therapy, Fabry disease, infusion‐associated reactions, infusion rate escalation protocol

## Abstract

**Background:**

Although enzyme replacement therapy with agalsidase beta resulted in a variety of clinical benefits, life‐long biweekly intravenous infusion may impact on patients’ quality of life. Moreover, regular infusions are time‐consuming: although a stepwise shortening of infusion duration is allowed up to a minimum of 1.5 hr, in most centers it remains ≥3 hr, and no data exists about the safety and tolerability of agalsidase beta administration at maximum tolerated infusion rate.

**Methods:**

In this study, we reported our experience with a stepwise infusion rate escalation protocol developed in our center in a cohort of 53 Fabry patients (both already receiving and treatment‐naΪve), and explored factors predictive for the infusion rate increase tolerability.

**Results:**

Fifty‐two patients (98%) reduced infusion duration ≤3 hr; of these, 38 (72%) even reached a duration ≤2 hr. We found a significant difference between the mean duration reached by already treated and naΪve patients (*p* < .01). More severely affected patients (male patients and those with lower enzyme activity) received longer infusions for higher risk of infusion‐associated reactions (IARs). A significant correlation between anti‐agalsidase antibodies and IARs was found.

**Conclusion:**

Our infusion rate escalation protocol is safe and could improve patient compliance, satisfaction and quality of life.

## Graphical abstract

Although enzyme replacement therapy with agalsidase beta resulted in a variety of clinical benefits, life‐long biweekly intravenous infusion may impact on patients’ quality of life because regular infusions are time‐consuming. In this study, we reported our experience with a stepwise infusion rate escalation protocol developed in our center in a cohort of 53 Fabry patients (both already receiving and treatment‐naΪve), and explored factors predictive for the infusion rate increase tolerability. We showed that our infusion rate escalation protocol is safe and could improve patient compliance, satisfaction and quality of life.

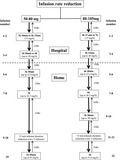

## 1 INTRODUCTION

Fabry disease (FD) is a rare, progressive multisystemic disease, caused by deficiency of the lysosomal enzyme alpha‐galactosidase A (αGal A) (Ortiz et al., 2018; Pisani et al., 2014). The current treatment options for FD include intravenous enzyme replacement therapy (ERT) (Eng et al., 2001; Schiffmann et al., 2001) and, more recently, an oral chaperone therapy (Germain et al., 2016; Riccio et al., 2020).

Two formulations for ERT have been commercially available in Europe for almost 20 years: agalsidase alfa (Replagal) and beta (Fabrazyme). Although these preparations are biochemically and structurally very similar (Blom et al., 2003; Lee et al., 2003; Sakuraba et al., 2006), there is a fivefold difference in recommended dose and, consequently, in the infusion duration (Fabrazyme® Prescribing Information, 2010; Replagal® Summary of Product Characteristics, 2014).

Although the use of ERT resulted in a variety of clinical benefits (Pieroni et al., 2021; Pisani et al., 2012), life‐long intravenous treatment with a every other week (eow) schedule, may interfere with daily activities and impact on quality of life. The possibility to transfer patients to home treatment after a first period of in‐hospital infusion has only partially improved patients’ satisfaction and quality of life (Smid et al., 2013), because regular infusions, especially with agalsidase beta, remain time‐consuming.

For the standard dose of Fabrazyme of 1 mg/kg body weight, the recommended initial intravenous infusion rate is no more than 0.25 mg/min (15 mg/hr) (Fabrazyme® Prescribing Information, 2010; SmPC, n.d.). Therefore, the initial infusion duration usually ranges from 3.5 to 7 hr based on body weight (for 50–105 kg), not including additional time needed for preparation of ERT, assessment of vital signs, intravenous access, and post‐infusion monitoring. This can be a burdensome time commitment for patients and their families in the long run.

As published in the U.S. prescribing information of Fabrazyme, after patient tolerance to the infusion is well established, an increase of the infusion rate of 0.05 to 0.08 mg/min (increments of 3 to 5 mg/hr) at every subsequent infusion is allowed, and the minimum infusion duration is 1.5 hr (based on individual patient tolerability) (Fabrazyme® Prescribing Information, 2010). On the contrary, despite the reduction of infusion time is also allowed by the European Summery of Product Characteristics (EMA SmPC) (n.d.), it is not specified how and when to reduce it.

Few studies have reported that patients were able to tolerate higher infusion rates and achieve shorter infusion times (≤2.5 hr) (Banikazemi et al., 2007; Pieroni et al., 2021), while some experiences with rapid intravenous infusions have defined a variable potential for hypersensitivity and anaphylactoid reactions, as well as various inflammatory and immunological responses (Milligan et al., 2006). On the whole, little is known about the effects of ERT administered at maximum tolerated infusion rate, and a stepwise infusion rate escalation protocol would be needed to safely reduce the infusion duration.

In 2006, we developed a stepwise infusion rate escalation protocol with the goal to shorten the infusion duration to the minimum tolerated, without compromising the safety and efficacy of the treatment.

In this study, we reported our experience with this ERT escalation protocol, and explored factors predictive for patient tolerability to the infusion rate increase.

## 2 METHODS

### 2.1 Study design

The present study is a monocentric, retrospective, observational study. The stepwise infusion rate escalation was applied to have an internal guideline to manage infusion in our everyday clinical practice, not as a research protocol.

All Fabry patients attending the Fabry Center of the Federico II University of Naples between September 2006 and November 2020, both already receiving and treatment‐naΪve to agalsidase beta at the approved dose, were considered for our infusion rate escalation protocol.

All clinical and laboratory data of interest were retrospectively gathered from patient clinical charts and electronic health records. In particular, all data were collected at time of protocol initiation (baseline); the development of neutralizing α‐GLA A antibodies (Ab status) was assessed by immunochromatographic (IC) assay, after protocol completion, when patients had already achieved the minimum tolerated duration, according to the method described elsewhere (Nakano et al., 2015).

### 2.2 Infusion rate escalation protocol

As published in the U.S. prescribing information of Fabrazyme, the recommended initial intravenous infusion rate is no more than 0.25 mg/min (15 mg/hr), and increments of the infusion rate of 0.05 to 0.08 mg/min (increments of 3 to 5 mg/hr) with each subsequent infusion are allowed, to a minimum infusion duration of 1.5 hr (Fabrazyme® Prescribing Information, 2010). Therefore, prior of the infusion rate escalation, all patients started with the recommended infusion rate of 15 mg/hr, with an infusion duration ranging from 3 hr 30 min for those receiving 50 mg to 7 hr for patients treated with 105 mg of agalsidase beta. Moreover, drug reconstitution was performed in accordance with the SmPC, and the total infused volume was 100 ml for patients weighing up to 70 kg, 250 ml for those of 70–100 kg, and 500 ml for patients weighing over 100 kg (Fabrazyme® Prescribing Information, 2010).

**FIGURE 1:**
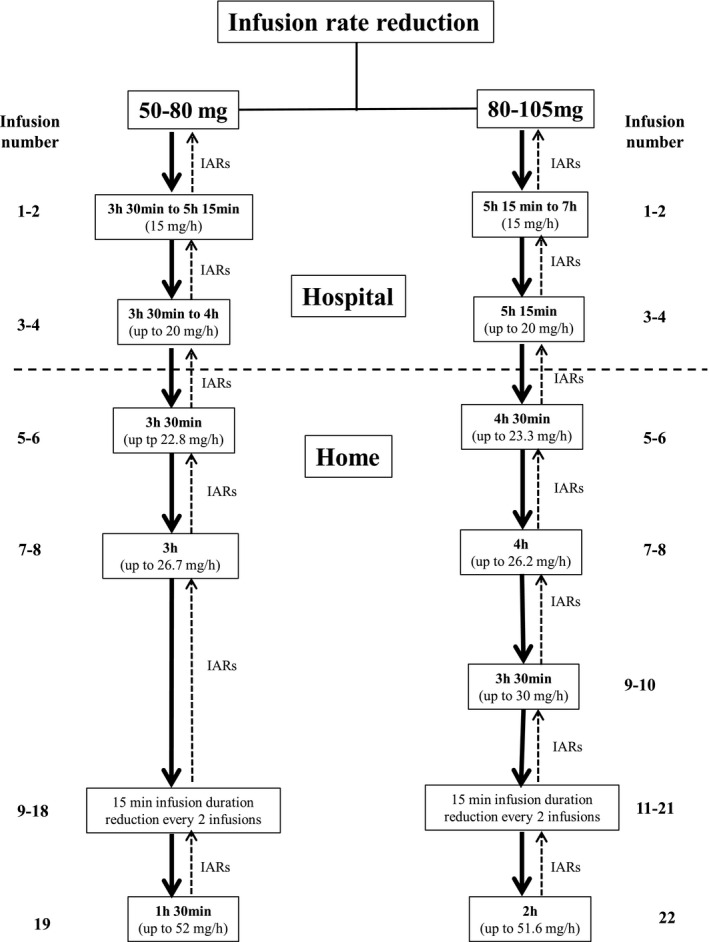
Escalated infusion rates and durations for patients with FD receiving agalsidase beta at the doses of 50–80 and 180–105 mg following our infusion rate escalation protocol

Once a patient tolerated the therapy, the infusion rate was increased by slowly upward titration, following a stepwise infusion rate escalation protocol, as shown in Figure 1. Each step consisted of reducing the infusion duration every other infusion. Rate adjustments were determined by the treating physician based on individual patient conditions. Patients proceeded to the next phase once tolerance to the increased infusion rate was established up to a minimum infusion duration of 1.5 hr. Moreover, we mandated patients to return to the previous step when they developed infusion‐associated reactions (IARs) and a maximum of two failed attempts to reduce the infusion duration were achieved.

Finally, since home therapy became available in our region, patients well tolerating the first four in‐hospital infusions were transferred home for the next infusions under nurse surveillance.

### 2.3 Infusion‐associated reactions assessment

Patients were monitored for IARs during each ERT infusion: specifically, all symptoms occurring during or up to 4 hr after ERT infusions were considered IARs. Patients experiencing IARs during the treatment needed to decrease the infusion rate and/or temporarily stop the infusion; moreover, for the subsequent infusion, pretreatment with an antipyretic and antihistamine drug was recommended, and the infusion rate remained constant (Linthorst et al., 2006; Smid et al., 2013). Serious IARs (SIARs) were IARs which needed medical treatment (i.e., antipyretics, antihistamines, or steroids); in addition, if anaphylactic or severe allergic reactions occurred during home therapy, patients returned to in‐hospital infusion since tolerance to ERT was established (Smid et al., 2013).

### 2.4 Statistical analysis

We explored correlation between infusion duration and sex, mutation, α‐GLA A activity, agalsidase beta dose, Ab status in all patients, and in the two subgroups of already treated and naΪve patients. Moreover, in patients experiencing IAR, we explored correlation with agalsidase beta dose received, sex, α‐GLA A activity, and Ab status.

All statistical analyses were performed in R (v3.6.1). Comparisons of paired data were performed by Wilcoxon test or paired *t*‐test for nonparametric and normally distributed data, respectively. Correlation analyses were performed by cor function (“stat” R package) and plotted by “corrplot” R package (v0.84). In the correlation analysis, the correlations concerning dichotomous variables were performed following the point‐biserial method. Analyses of the frequency tables formed by categorical variables were performed by a chi‐square test of independence (*χ* ^2^ test function of “stat” R package). All figures were built with “ggplot2” R package (v3.3.2). Level of significance for all analyses was *p* < .05.

## 3 RESULTS

Of the 56 patients who received ERT with agalsidase beta at the approved dose following our infusion rate escalation protocol between September 2006 and November 2020, 53 [28 (53%) males] completed the protocol‐required period and were included in our analysis.

The main characteristics at baseline of the overall population included in the study, and in the two subgroups of already treated and naΪve patients, are shown in Table 1.

**TABLE 1:** Baseline characteristics of enrolled patients

	All patients (N = 53)	Already in ERT (N = 11)	NaΪve (N = 42)
Age (years)	46.75 ± 14.44	50.36 ± 11.31	45.81 ± 15.13
Sex (M/F, N)	28/25	7/4	21/21
αGLA activity (nmol/hr/ml)	2.07 ± 2.18	0.96 ± 1.29	2.36 ± 2.29*
GLA variant (classic/late‐onset, N)	38/15	11/0	27/15*
Agalsidase beta dose (mg)	77.9 ± 14.6	79.5 ± 15.6	77.5 ± 14.4
FD‐related manifestations (N, %)			
Cardiac	18 (34)	3 (27)	15 (36)
Renal	20 (38)	4 (36)	16 (38)
Cerebrovascular	5 (9)	1 (9)	4 (9)
Other	19 (36)	3 (27)	16 (38)

Note

Data are expressed as mean ± *SD* or number and percentage.

Abbreviations: ERT, enzyme replacement therapy; F, female; FD, Fabry disease; M, male.

* *p* < .05 versus already treated.

Of the 53 analyzed patients, 11 started our escalation protocol in September 2006, after a mean period of 40.91 ± 17.76 months of ERT with agalsidase beta; the other 42 patients were treatment‐naΪve, and started ERT de novo following our protocol. Patients of the two groups were comparable for age, sex, and agalsidase beta dose, while already treated patients had more classic GLA mutations and lower αGal A activity when compared to naΪve patients. None of the patients had received a kidney transplant or used any corticosteroids during the protocol period.

All patients followed our infusion rate escalation protocol, as reported in Figure 1. Patients proceeded to the next phase once tolerance to the increased infusion rate was established, and they continued with the minimum tolerated infusion duration achieved. Since home therapy became available in our region, all patients were transferred to nurse‐supervised home treatment after the first four in‐hospital infusions.

**FIGURE 2:**
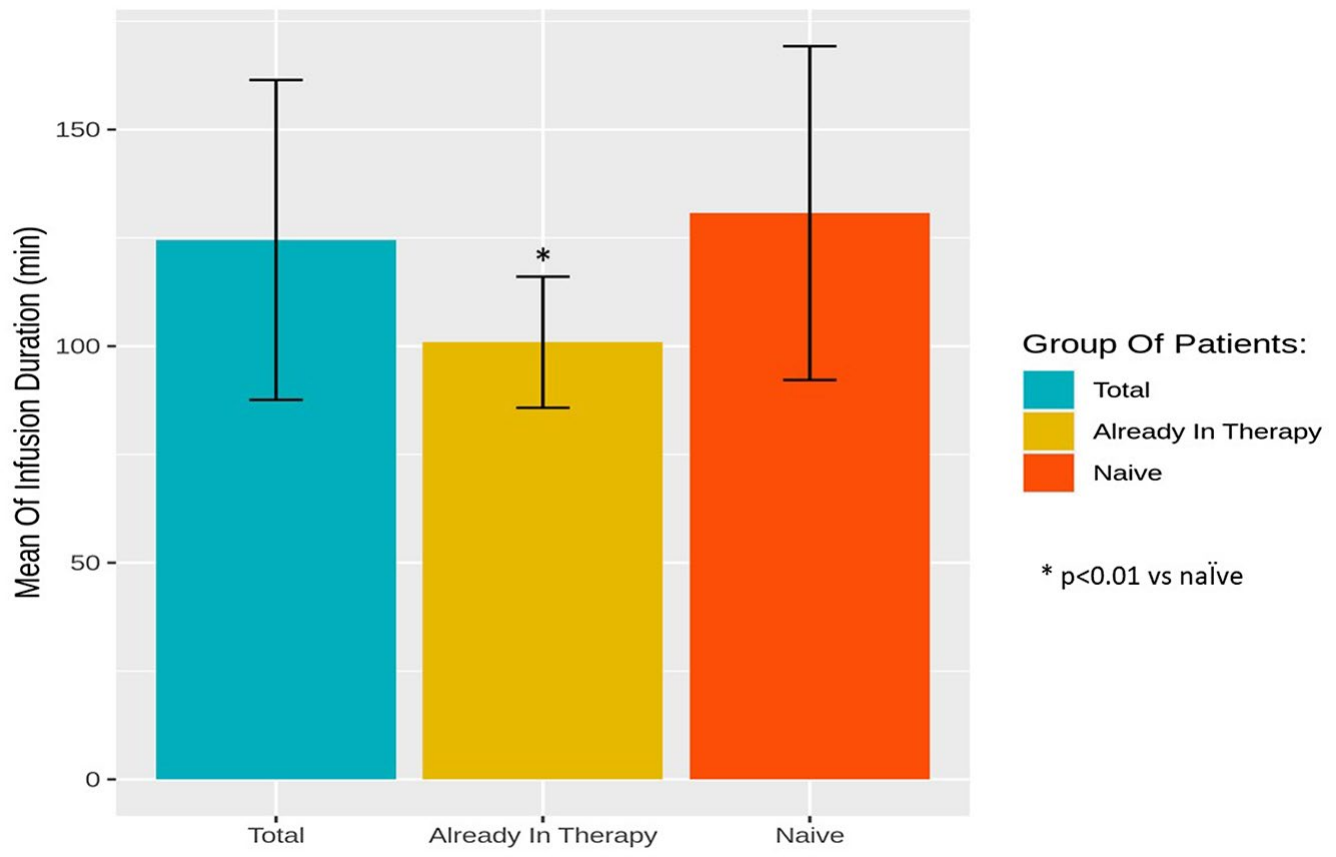
Mean minimum infusion duration reached in total group, and in the two subgroups of patients already in ERT and naΪve

The 53 analyzed patients reached a mean infusion duration of 124.53 ± 36.93 min, with a significant difference between already treated and naΪve patients (100.91 ± 15.14 vs. 130.71 ± 38.53 min, *p* < .01; Figure 2).

**FIGURE 3:**
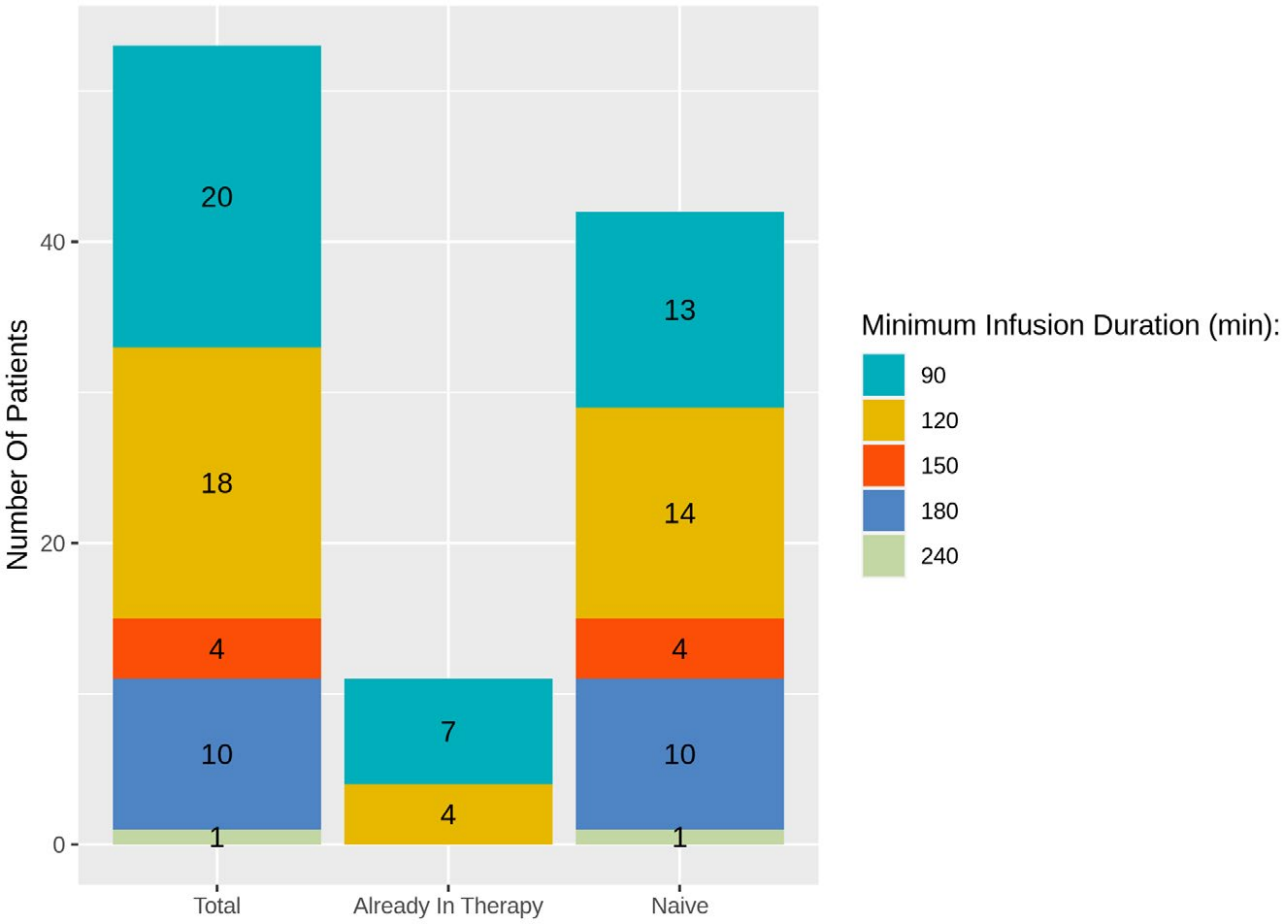
Distribution of patients, of total group and of the two subgroups already in ERT and naΪve for each minimum infusion duration achieved

With the exception of a single patient with difficulties to receive intravenous fluid infusions for a severe cardiovascular disease, all patients (98%) successfully reduced infusion duration ≤3 hr; of these, 38 patients (72%) even reached an infusion duration ≤2 hr. The details of patients of each infusion duration group are reported in Figure 3 and Table 2.

**TABLE 2:** Characteristics of patients of each infusion duration group

	90 min	120 min	150 min	180 min	240 min
	(N = 20)	(N = 18)	(N = 4)	(N = 10)	(N = 1)
State of treatment					
Already in ERT	7	4	0	0	0
NaΪve	13	14	4	10	1
Sex					
Male	7	9	2	9	1
Female	13	9	2	1	0
GLA variant					
Classic	18	10	3	6	1
Late‐onset	2	8	1	4	0
Agalsidase beta dose					
50–80 mg	16	15	4	5	1
80–105 mg	4	3	0	5	0
αGLA A (nmol/hr/ml)	3.1 ± 2.6	1.45 ± 1.52	2.27 ± 1.45	1.17 ± 1.47	0.9
Anti‐agalsidase Ab					
Available/not	6/14	3/15	1/3	8/2	0/1
Positive	1	2	1	7	0
Negative	5	1	0	1	0
IAR during protocol	0	15	4	10	1
IAR post protocol	3	4	1	5	1

Note

Data are expressed as number or mean ± *SD*.

Abbreviations: Ab, antibodies; ERT, enzyme replacement therapy; IAR, infusion adverse reaction.

Therefore, a total of 23 patients (43.4%) successfully reached the minimum infusion duration of 90 min (20 patients, 16 following the protocol for the dosage of 50–80 mg and 4 following that for the dosage of 80–105), or 120 min (3 patients receiving 80–105 mg of agalsidase beta).

**FIGURE 4:**
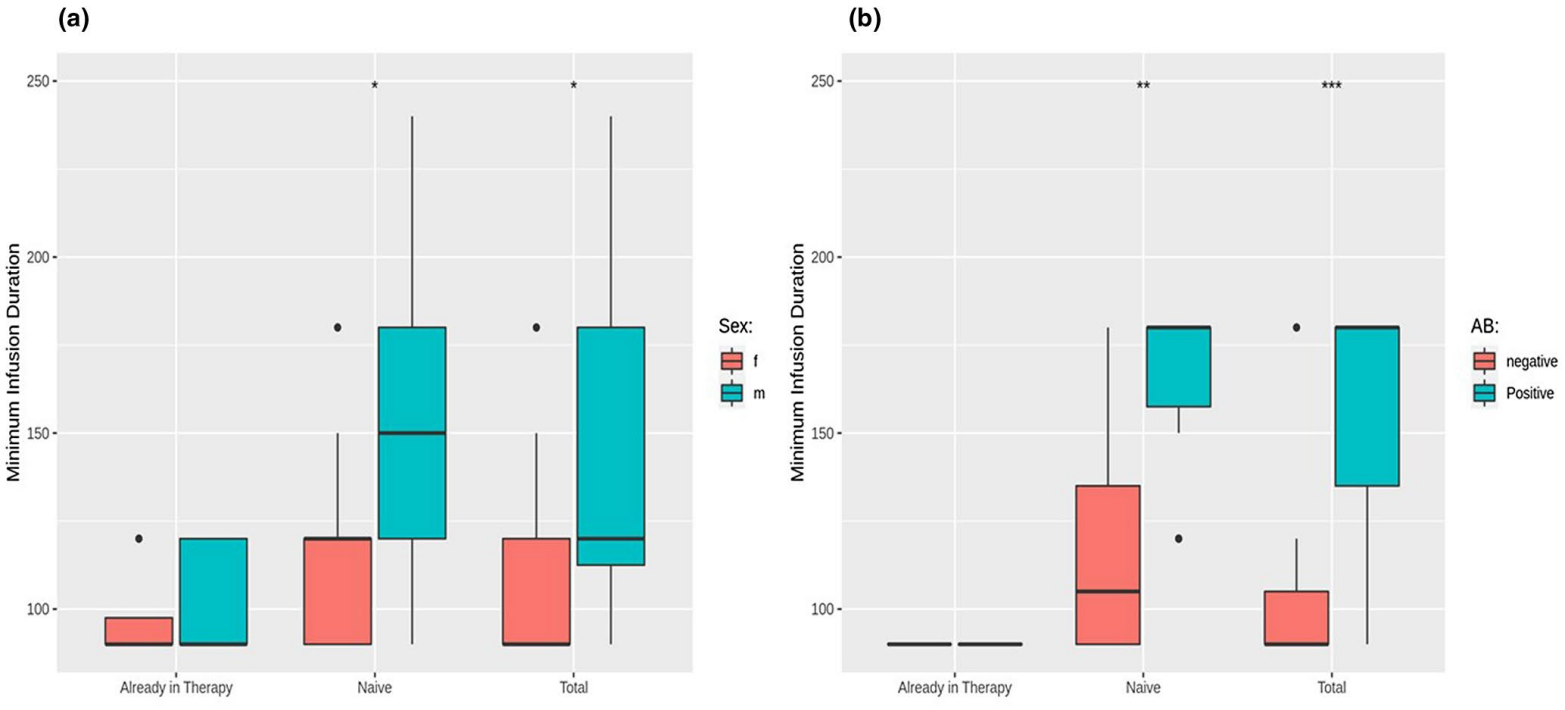
The box‐plots show the correlation between categorical feature sex (a), and antibodies development (a,b) and minimum infusion duration in total, already treated, and naΪve patients. Chi‐squared contingency table tests were performed using a minimum infusion duration of 120 to split the patients. *p < .05, **p > .05, ***p = .05

**FIGURE 5:**
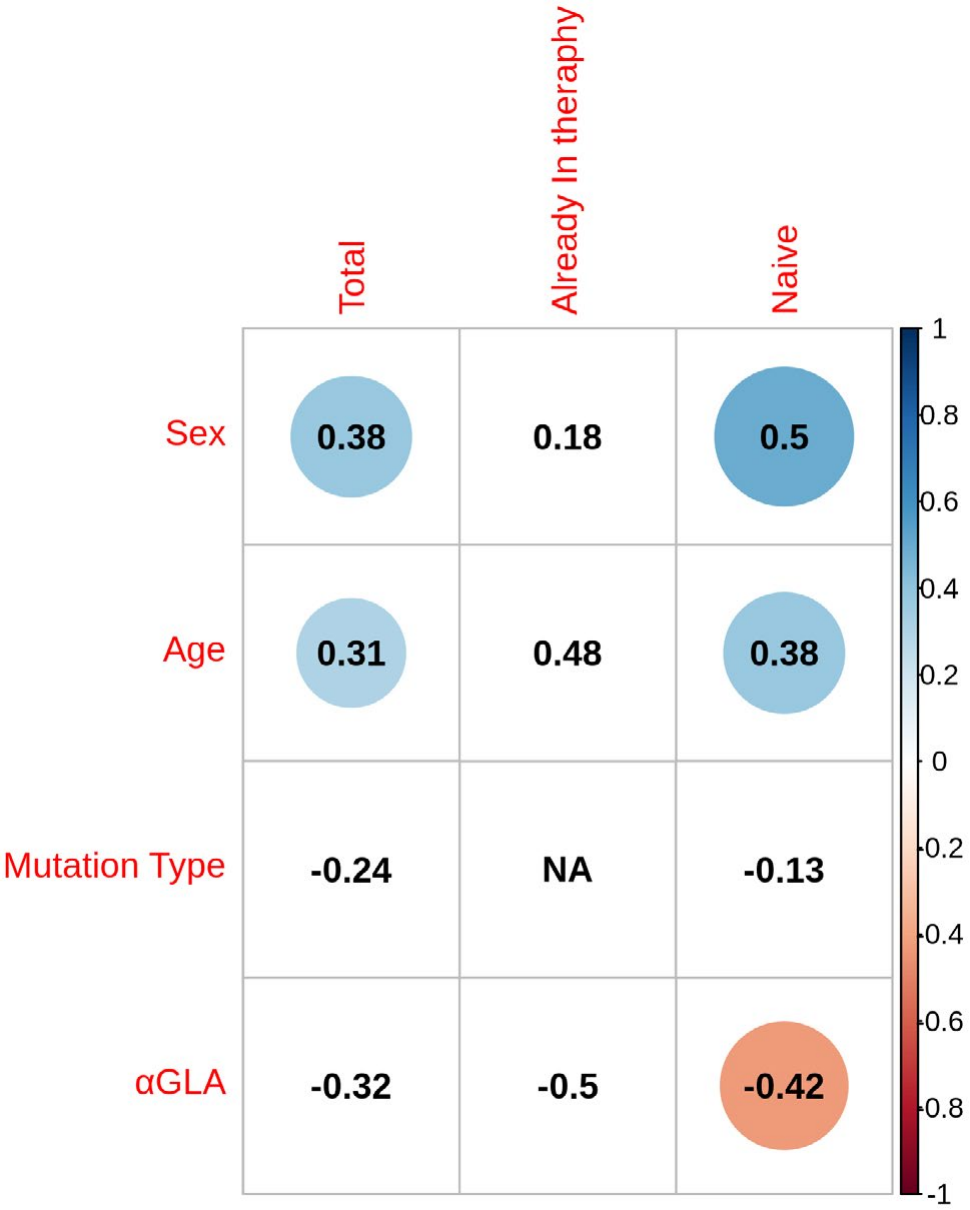
Correlation plot between age, sex, mutation type, and α‐GLA A activity and the infusion duration in total, already treated, and naΪve patients. Circled values are significant correlations (*p* < .05): blue indicates a positive correlation and red a negative correlation

As shown in Figures 4 and 5, the infusion duration was longer in male patients in total and naΪve groups and in patients with antibodies in total group. Moreover, in naΪve patients, we found a negative correlation between infusion duration and αGal A activity (Figure 5). No correlation was found with mutation type (Figure 5) and agalsidase beta dose (data not shown).

Thirty patients (56.6%) not tolerating infusion duration reduction to the minimum of 90 min, developed IARs when infusion rate was increased, and continued to receive ERT at the maximum tolerated infusion rate. More specifically, 18 reported dizziness (33% of the total), 12 gastrointestinal symptoms (abdominal pain, nausea and/or vomiting, diarrhea) (22.6%), 10 headache (18.8%), 6 pain in extremities (11.4%), 5 dyspnea (9.4%), 4 cough (7.6%), 3 nasopharyngitis (5.7%), 3 fever (5.7%), and 1 dry mouth (1.9%). All IARs were mild or moderate in intensity, and were managed with premedication at the following infusions. Only three patients reported SIARs, needing administration of antipyretics, antihistamines, and/or steroids to manage the reactions. Finally, none of them were considered to be serious adverse events.

Moreover, 14 patients reported IARs after protocol completion (after a mean period of 75.3 ± 61.27 months). Specifically, three patients were receiving ERT in 90 min (one male, already treated, Ab negative, reported chills; two females, naΪve, with antibodies not available, reported headache); four in 120 min (three naΪve males and one already treated female, all reporting chills and with not available antibodies); one naΪve male, positive to antibodies, receiving therapy in 150 min, reported chills; five patients were treated in 180 min (all naΪve males, positive to antibodies, feeling chills, one also fever, one increase in arterial blood pressure and headache); finally, one naΪve male patient treated in 240 min, reported chills and dyspnea despite premedication. All IARs were transient and of mild intensity, and usually spontaneously resolved within 2 hr.

The development of IARs was higher in males, in patients with low αGal A activity and in those with antibodies in the group of total and naΪve patients, while mutation type and received dose were not associated with the development of IARs (Table 3).

**TABLE 3:** Factors associated with development of infusion‐associated reactions

	Total IARs (N = 30)	IARs in already treated group (N = 3)	IARs in naΪve group (N = 27)
Infusion duration			
90 min	0	0	0
120 min	15	3	12
150 min	4	0	4
180 min	10	0	10
240 min	1	0	1
Sex			
Male	20*	2	18*
Female	10	1	9
α‐Gal A activity (nmol/hr/ml)	1.4 ± 1.5**	0.3 ± 0.3	1.5 ± 1.5**
Agalsidase beta dose			
50–80 mg	25	3	22
80–105 mg	5	0	5
Ab status			
Positive	10*	0	10*
Negative	2	0	2
Not available	18	3	15
GLA variant			
Classic	17	3	14
Late‐onset	13	0	13

Abbreviations: Ab, antibody; IARs, infusion‐associated reactions.

* Positive correlation (*p* < .05).

** Negative correlation (*p* < .05).

During the study period, one male patient receiving therapy in 2 hr for 49 months died at the age of 55 years with sudden cardiac death, and one male patient aged 58 started hemodialysis after 49 months of ERT infused in 3 hr.

Moreover, 12 patients discontinued agalsidase beta after a mean period of 3.9 years of therapy with the minimum tolerated infusion duration. In details: five of them were receiving treatment in 90 min (one switched to galafold for choice, one to investigational drug for participation in a clinical trial, and three to agalsidase alfa for shortage of beta); two patients were treated in 120 min (one switched to galafold for choice and one to alfa for shortage); and five were treated in 180 min (one for participation in a clinical trial, and four switched for IARs, of whom two to alfa and two to galafold).

## 4 DISCUSSION

This study, based on long‐term experience of a large Fabry cohort, showed for the first time that agalsidase beta administered at the minimum tolerated duration following our infusion rate escalation protocol is safe and well tolerated.

The initial infusion duration usually ranges from 3 hr 30 min for patients treated with 50 mg of agalsidase beta to 7 hr for those receiving 105 mg, when using the recommended infusion rates, and a stepwise shortening of infusion duration is allowed to a minimum of 1.5 hr, based on individual patient tolerability (Fabrazyme® Prescribing Information, 2010). However, a recent Italian survey showed that 75% of centers were not aware of the possibility to increase infusion rate, and the average infusion duration in most centers remained ≥3 hr (data not published).

To date, no study has evaluated the effects, safety and tolerability of agalsidase beta administered at maximum tolerated infusion rate, and an infusion rate escalation protocol to safely reduce infusion time does not exist.

Few studies have reported that patients were able to tolerate higher infusion rates. In particular, Germain et al. reported that in a study population of 44 Fabry patients treated with agalsidase beta for a study period of 54 months, the infusion time had decreased from 4 to 6 hr at the start of the study to a mean 2.5 hr, with 72% of patients completing the majority of their infusions in ≤2.5 hr and 48% completing most of their infusions in ≤2 hr (Pieroni et al., 2021). Moreover, Banikazemi et al. reported data on 51 patients treated with agalsidase beta at the initial recommended rate of 0.25 mg/min, which was increased after the eighth infusion to decrease the infusion time to a minimum of 90 min. All patients were pretreated with acetaminophen or ibuprofen and some patients with an antihistamine to minimize IARs (Banikazemi et al., 2007). Other papers reported that rapid intravenous infusions could be responsible for hypersensitivity and anaphylactoid reactions as well as various inflammatory and immunological responses, particularly in‐home environment (Milligan et al., 2006; Smid et al., 2013).

In this study, we reported our experience with a stepwise infusion rate escalation protocol developed in our center in 2006 with the aim to safely reduce infusion duration to the minimum tolerated, in a cohort of 53 patients with FD. All analyzed patients (98%) successfully reduced infusion duration ≤3 hr, with the exception of a single one with difficulties to receive intravenous fluid infusions for a severe cardiovascular disease; of these, 38 patients (72%) even reached an infusion duration ≤2 hr. Interestingly, we found a significant difference between the mean duration reached by already treated and naΪve patients (100.91 ± 15.14 vs. 130.71 ± 38.53 min, *p* < .01), consistent with the evidence that most IARs tend to decrease in severity as the duration of therapy increases. In fact, it has been widely reported that most IARs to ERT occur in the first few months of treatment (2–4 months), regardless of infusion duration (Linhart et al., 2020; Smid et al., 2013). Moreover, they have been largely correlated with the presence of αGal A antibodies (Wanner et al., 2018), though negative antibody status does not preclude infusion reactions, and patients usually develop these antibodies during the first 6 months of treatment (the majority within 3 months) (Eng et al., 2001; Lenders & Brand, 2018; Schiffmann et al., 2001). Therefore, we believe that the stepwise infusion rate escalation suggested by our protocol, that reaches the minimum infusion duration in a period of 19 weeks (9 months), is sufficient for safe treatment.

As expected, our results confirmed the significant correlation between that anti‐agalsidase antibodies and the occurrence of IARs. However, although the risk of developing IARs is largely based on ab status, its measurement was not implemented in our protocol for practice purposes, and data were assessed in only 18 patients after protocol completion, when they had already achieved the minimum tolerated infusion duration. Of these 18 patients with known Ab status, 10 Ab‐positive and 2 Ab‐negative experienced IARs during the infusion rate escalation; surprisingly, one Ab‐positive patient receiving agalsidase beta 105 mg well tolerated the escalation to the minimum infusion duration of 90 min. Therefore, Ab status is not fully predictive for the occurrence of IARs, as correlation was tested only in patients with available data on Ab status.

Similarly, our results showed that more severely affected Fabry patients (e.g., male patients and those with lower αGal A activity) were at higher risk of developing IARs during protocol, and therefore, received infusions with longer duration. But again, these factors were not fully predictive of IAR occurrence, as severity scores were not assessed and no correlation was found between IARs and mutation type.

IARs did not occur during the following infusions when patients received premedication, as indicated in‐home treatment algorithm for FD (Linthorst et al., 2006; Smid et al., 2013). Therefore, only for patients developing IARs, pretreatment with an antipyretic and antihistamine was recommended for subsequent infusion, and infusion rate remained constant.

Our study showed that the frequency of IARs did not increase during the infusion rate escalation protocol: the number of IARs and adverse events recorded during the protocol, that were not higher than expected, all of mild intensity and managed at home without recourse to hospital, clearly demonstrated that our protocol can be considered safe and well tolerated. Moreover, infusion rate escalation can be safely achieved in‐home infusions settings as most of our patients were receiving home infusion during the protocol (39 patients, 74%).

Though patient compliance and satisfaction were not specifically addressed, reduction of infusion duration has substantial positive effects on both issues, as has been demonstrated in other lysosomal storage disorders (Desai et al., 2018).

Our study was limited by the retrospective nature, relying on medical records, reports of home‐care service, data collected in Fabry registry, and patient narratives during outpatient clinic appointments.

Further studies with prospective design are needed to confirm the safety and efficacy of this infusion rate escalation protocol, to evaluate the impact on patient compliance, satisfaction, and quality of life, and to correctly assess the correlation between the increase of infusion rate, development of antibodies and occurrence of IARs.

We conclude the stepwise shortening of infusion duration to the minimum tolerated following our infusion rate escalation protocol in eligible Fabry patients is safe and could contribute to improve treatment compliance, patient satisfaction, and self‐perceived quality of life.

## ETHICAL COMPLIANCE

All patients provided written informed consent. The study was conducted in accordance with the Declaration of Helsinki and was approved by the institutional ethics committee.

## CONFLICT OF INTEREST

The authors declare that no conflict of interest exists.

## AUTHOR CONTRIBUTIONS

Conceptualization: A.P., E.R.; Data curation: E.R., M.Z., M.F., I.C., P.B.; Formal Analysis: M.Z., M.F., P.B.; Investigation: A.P., E.R., I.C., M.A., L.F.; Methodology: M.Z., M.F.; Project administration: A.P.; Resources: A.P.; Software: M.A., I.C., L.F.; Supervision: A.P., E.R.; Validation: A.P., E.R.; Writing – original draft: E.R.; Writing – review & editing: E.R., A.P., M.Z.

## Data Availability

The data that support the findings of this study are available from the corresponding author upon reasonable request.
